# How evidence-based workforce planning in Australia is informing policy development in the retention and distribution of the health workforce

**DOI:** 10.1186/1478-4491-12-7

**Published:** 2014-02-03

**Authors:** Ian F Crettenden, Maureen V McCarty, Bethany J Fenech, Troy Heywood, Michelle C Taitz, Sam Tudman

**Affiliations:** 1 Health Workforce Australia, 400 King William Street, Adelaide, Australia

**Keywords:** Workforce planning, Workforce projections

## Abstract

**Background:**

Australia’s health workforce is facing significant challenges now and into the future. Health Workforce Australia (HWA) was established by the Council of Australian Governments as the national agency to progress health workforce reform to address the challenges of providing a skilled, innovative and flexible health workforce in Australia. HWA developed Australia’s first major, long-term national workforce projections for doctors, nurses and midwives over a planning horizon to 2025 (called Health Workforce 2025; HW 2025), which provided a national platform for developing policies to help ensure Australia’s health workforce meets the community’s needs.

**Methods:**

A review of existing workforce planning methodologies, in concert with the project brief and an examination of data availability, identified that the best fit-for-purpose workforce planning methodology was the stock and flow model for estimating workforce supply and the utilisation method for estimating workforce demand. Scenario modelling was conducted to explore the implications of possible alternative futures, and to demonstrate the sensitivity of the model to various input parameters. Extensive consultation was conducted to test the methodology, data and assumptions used, and also influenced the scenarios selected for modelling. Additionally, a number of other key principles were adopted in developing HW 2025 to ensure the workforce projections were robust and able to be applied nationally.

**Results:**

The findings from HW 2025 highlighted that a ‘business as usual’ approach to Australia’s health workforce is not sustainable over the next 10 years, with a need for co-ordinated, long-term reforms by government, professions and the higher education and training sector for a sustainable and affordable health workforce. The main policy levers identified to achieve change were innovation and reform, immigration, training capacity and efficiency and workforce distribution.

**Conclusion:**

While HW 2025 has provided a national platform for health workforce policy development, it is not a one-off project. It is an ongoing process where HWA will continue to develop and improve health workforce projections incorporating data and methodology improvements to support incremental health workforce changes.

## Background

### Challenges facing Australia’s health workforce

The following significant challenges are facing Australia’s health workforce now and into the future.

#### The self-sufficiency challenge

Australia has a high level of dependence on internationally recruited health professionals relative to most other Organisation for Economic Co-operation and Development countries [1], particularly for doctors. A number of other developed countries are in the same situation as Australia, and it is likely that its reliance will come under challenge as international competition for health workers increases.

#### The demographic challenge

Australia's population is ageing. Impacts of this include fewer working age people available to support older Australians; increasing losses from the health workforce as the current health workforce ages; a smaller pool of working age people from which we can draw our future health workforce; and a larger pool of older Australians who will consume more health care services. These challenges are compounded by the changing burden of disease in the community with an increasing prevalence of chronic conditions such as diabetes.

#### The cost challenge

Evidence suggests the health workforce accounts for approximately 70% of health care costs [2,3]. As demand for health services is expected to increase due to demographic changes, the cost of maintaining current levels of activity will increase - indicated by projections showing that Australian expenditure on health and residential aged care as a percentage of gross domestic product could rise from 9.3% in 2002/2003 to 12.4% by 2032/2033 [4].

#### The co-ordination challenge

Australia’s health care system, in a federated country^a^, is complex, with different levels of government responsible for funding, service provision and education and training, making it difficult to adopt a co-ordinated approach to planning for, and responding to, workforce issues.

#### The distribution challenge

Australia is geographically vast, and access to health professionals, particularly in rural and remote areas, is a significant issue that will likely be exacerbated as the demographic challenges outlined above take effect in the future.

#### The challenge of implementing workforce reform

Substantial barriers exist to implementing health workforce innovation or reform to improve workforce productivity, including the co-ordination challenge already highlighted, along with additional barriers such as legislation, organisation culture, resourcing, leadership and existing models of care and associated incentives.

### National health workforce planning and Health Workforce Australia

Many of the outlined challenges have existed for a number of years and, in recognition of this, health workforce planning has existed in Australia for many years. In 1995 the Australian Medical Workforce Advisory Committee (AMWAC) was established, to “assist with the development of a more strategic focus on medical workforce planning in Australia” [5]. In 2000, the Australian Health Workforce Advisory Committee (AHWAC) was established to oversee national level, government initiated health workforce planning for the nursing, midwifery and allied health workforces. AMWAC and AHWAC ceased in June 2006; however, at the same time the Council of Australian Governments^b^ (COAG) agreed to a significant national health workforce reform package which included the establishment of the National Health Workforce Taskforce, which was a time-limited entity (ceasing on 30 June 2010). Each of these organisations carried out national health workforce planning. However the need to link higher education and workforce was recognised, and in 2008 COAG agreed to the National Partnership Agreement on Hospital and Health Reform. This acknowledged that a national, co-ordinated approach to health workforce reform was necessary with a particular focus on linking efforts of health and higher education sectors. Subsequently, Health Workforce Australia (HWA) was established as the national agency to progress health workforce reform and address the challenges of providing a skilled, innovative and flexible health workforce. HWA is an Australian Commonwealth statutory authority and reports to the Standing Council on Health^c^ (SCoH).

SCoH commissioned HWA to undertake a workforce planning exercise for doctors, nurses and midwives over a planning horizon to 2025. The objective was to present and measure possible future health workforce outcomes under a range of workforce planning scenarios, and was titled Health Workforce 2025 (HW 2025).

### Purpose of the Health Workforce 2025 project

The outlined challenges have substantial implications for the ability of Australia’s health workforce to meet future health needs. The challenges are national in nature and so HW 2025 was primarily focussed at the national level. National planning allows a single, consistent approach to the management of the workforces. It is also only at the national level that questions of aggregate supply and demand can be separated from issues of allocation and distribution - the principal aim being to ensure an appropriate pool of professionals is available to meet aggregate demand.

By providing long-term, national workforce projections and presenting the best available planning information on Australia’s future medical, nursing and midwifery workforces, the HW 2025 project provided a platform for nationwide discussions on future workforce policy and reform directions, to build a sustainable health workforce for Australia.

### Purpose of this paper

This paper demonstrates how evidence-based workforce planning is being used in Australia to inform effective policy development. It presents the methodologies and underlying principles used by HWA in the HW 2025 project, summary results of the workforce planning projections, and the actions being taken to respond to the findings of the workforce projections.

## Methods

Health workforce planning is conducted across many countries using different methodologies. Many workforce planning models focus on using demographic trends to assess future supply and demand; others try to link health expenditure projections with health workforce projections; some take into account role extension and substitution; while others are trying to move beyond health service utilisation to needs-based models, as well as some examining multi-professional groups rather than professional groups in isolation [6,7].

A review of existing workforce planning methodologies, in concert with the project brief to HWA (to undertake a workforce planning exercise for doctors, nurses and midwives to present and measure possible future health workforce outcomes under a range of workforce planning scenarios), and an examination of data availability, identified the methodology outlined below as the best fit-for-purpose for the HW 2025 project.

### Estimating workforce supply

HW 2025 used a dynamic stock and flow model to estimate future workforce supply at a national level in Australia. The four key inputs in the HW 2025 dynamic stock and flow model were: 1) workforce stock (in 5-year age and gender cohorts); 2) domestic new entrants; 3) migration (permanent and temporary); and 4) net exits, which included all permanent and temporary flows out of the workforce.

In the stock and flow method, the number and characteristics of the current workforce (stock) are identified, along with the sources and number of workforce inflows and outflows. Trends or influences impacting on the stock and flows are also identified.

To project future supply, the initial workforce stock is moved forward based on expected inflows and outflows, allowing for the impact of identified trends and influences on the stock.

In the dynamic stock and flow model, the effect of people ageing is also accounted for. The workforce stock is broken down into age and gender cohorts, and each cohort receives inflows not just from graduates and migration (external flows), but also from people moving from one age cohort into the next. Similarly, each age and gender cohort has exits applied - from people leaving the workforce altogether, as well as exits as a person moves into the next age cohort. This is an iterative calculation for each year over the projection period, and provides for a more realistic representation of labour market dynamics.

**Figure 1:**
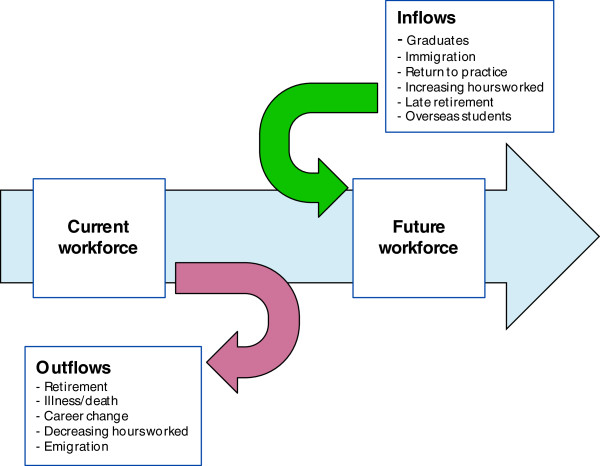
Stock and flow process.

The stock and flow process is represented in Figure 1, where people entering and exiting the workforce (the flows) periodically adjust the initial number in the workforce stock to project future supply.

### Estimating workforce demand

HWA employed the utilisation method to develop workforce demand projections. This approach measures expressed demand, and is based on service utilisation patterns as they currently exist. It makes no assumptions about potential demand, or unmet demand.

Service utilisation data were matched against age and gender cohorts and, once mapped, were projected against future demographic structures. Mapping service utilisation to age and gender cohorts captures changes in service utilisation associated with changes in population composition. For example, if a particular set of services is associated with 35- to 39-year-old females and their share of the overall population increases, then demand for the workforces associated with the provision of those services will grow greater than the rate of the overall population.

In HW 2025, unique expressed demand growth rates were calculated for each medical specialty, nursing area of practice, and midwifery.

Key data sets used to generate the HW 2025 workforce supply and demand projections are presented in Table 1.

**Table 1:** Key national data sets/sources

**Variable**	**Data source**
**Doctors**	
Workforce headcount/demographics	AIHW medical labour force survey
Graduates	Medical Deans Australia and New Zealand
Fellows	Medical colleges
Immigration	Department of Immigration and Border Protection
Demand	Hospital separation statistics
	Medicare utilisation statistics
	Australia New Zealand Intensive Care Society
**Nurses**	
Workforce headcount/demographics	AIHW nursing and midwifery labour force survey
Graduates	Department of Education (for registered nurses) and NCVER (for enrolled nurses)
Immigration	Department of Immigration and Border Protection
Demand	Hospital separation statistics
	Community care places
	Residential high care places
	Home and community care data
	Australia New Zealand Intensive Care Society
**Midwives**	
Workforce headcount/demographics	AIHW nursing and midwifery labour force survey
Graduates	Department of Education
Immigration	Department of Immigration and Border Protection
Demand	ABS Australian population projections series B

*ABS*, Australian Bureau of Statistics; *AIHW*, Australian Institute of Health and Welfare; *NCVER*, National Centre for Vocational Education Research.

### Scenario analysis

Scenario analysis was used to demonstrate the impact of potential policy options on future workforce supply and demand. The method used was to present a comparison scenario, where current trends in supply and expressed demand were assumed to continue into the future, and use this to compare with a range of alternative scenarios. Varying input parameters in the workforce projection model generated the alternative scenarios. The flow through effect to the future workforce was then measured through the impact relative to the comparison scenario. The alternative planning scenarios were categorised according to the policy options they fit within, and included the following. (Not all scenarios that were modelled are listed in this article. Full details of all scenarios are contained in the HW 2025 suite of publications.)

#### Innovation and reform scenarios

##### Productivity scenario

The demand for the workforce was reduced at a notional rate of 5% over the projection period, to illustrate productivity improvements through reforms including changed skill mix, changing models of care, technological change or other reforms.

##### Low demand scenario

The demand for the workforce was reduced by a notional value of two percentage points.

##### Workforce retention scenario (nurses only)

The supply of the nursing workforce was increased through improvements in the nursing retention rate.

#### Immigration scenarios

##### Medium and high self-sufficiency scenarios

Immigration was progressively reduced to 50% and 95% of starting levels, respectively, to show the relative reliance of the workforce on international health professionals.

#### Other impact scenarios

##### High demand scenario

The demand for the workforce was increased by a notional value of two percentage points.

##### Capped working hours scenario (doctors only)

Capped the total number of hours worked by the total medical workforce at a notional value of 50 hours per week, to demonstrate the effect of a reduction of working hours for all doctors.

The scenarios were not used as predictions of the future, but were used to provide an estimate of a likely outcome given the set of conditions and assumptions upon which the scenario was based.

### Principles underlying the methodology

In developing the HW 2025 project, HWA followed a number of key principles to ensure the workforce projections generated were robust and realistic [8], and able to be used as a framework for nationwide discussions on future workforce policy and reform directions.

#### Methodological robustness and coherency

The selection of the workforce projection methodologies used involved consideration of a broad range of literature relating to health workforce planning and modelling [9-13]. The methodology chosen (described above) was determined to be the most fit-for-purpose, and was applied across the medical, nursing and midwifery workforces. This consistency and coherency in application allowed for meaningful comparisons and policy considerations at a national level.

#### Use of national data

All input data was sourced from nationally comparable data sets (Table 1). This meant the characteristics of the existing workforces and derived items such as exit rates were all developed on the same basis across Australia. The use of national data reinforced the coherence and consistency of applying the same methodology across workforces to allow for meaningful national comparisons.

#### Explicit assumptions

Workforce projections provide likely outcomes given the assumptions on which they are based. The assumptions underpinning HW 2025 were exposed for critical review through an extensive consultation process to ensure they were realistic and defensible. The underpinning assumptions were also published with the workforce projections to ensure the results could be interpreted accurately.

#### Consultation and review processes

The methodology, data and underpinning assumptions that created the HW 2025 workforce projections were consulted on extensively through the course of the project. In particular:

• A Technical Reference Group, composed of representatives from academia, government and the health sector, provided advice and expertise on issues including the appropriateness of the underpinning assumptions and best practice approaches to quantifying education and training capacity and modelling workload measures.

• The methodology paper was available for public comment.

• Structured workshops were conducted with workforce participants and organisations to expose the overall method and the assumptions underlying the baseline projections to critical review.

• Clinical leads (health professionals representing each of the fields of medicine, nursing and midwifery) provided clinical expertise and context to the workforce projections and the development of alternative scenarios.

#### Iterative process

Workforce projections become less accurate as the period of time over which they apply increases. The World Health Organization noted “It is therefore critical that plans include mechanisms for adjustment according to changing ongoing circumstances. Making projections is a policy-making necessity, but is also one that must be accompanied by regular re-evaluation and adjustment” [9]. HW 2025 projections will be updated as new data become available, and the methodology and assumptions will be periodically reviewed with the assistance of clinical experts to ensure the projections remain realistic and relevant.

### Value of the Health Workforce 2025 methodology and principles

As outlined earlier, health workforce planning can be conducted using different methodologies. Many institutions in Australia, including state and territory governments, employers, professions and other planners, also conduct health workforce planning. Such workforce planning is often conducted for different purposes and has different scopes, data sources and assumptions. A national picture from such workforce planning cannot be obtained.

While previous national health workforce planning has also been conducted, this was in a siloed approach - examining individual specialty workforces (for example, anaesthesia specialists, radiology specialists, critical care nurses) in isolation and at different points in time.

Historically, there has also been no connection between the health and higher education sectors when conducting workforce planning, which is important given the vital role the education sector plays in generating the future health workforce.

HW 2025 addresses the above limitations. Using the methodology and principles outlined, HWA has developed a set of nationally authoritative, consistent and coherent health workforce projections to be used for health workforce planning. The national nature of the workforce projections is vital. National challenges are facing the health workforce, and the national planning conducted allows, for the first time, a single consistent approach to workforce management.

HW 2025 provides the evidence base from which student and training intakes can be aligned with projected health workforce requirements. As part of this, HWA has a responsibility to develop and implement programmes to increase the capacity and effectiveness of clinical training for health professions - providing a clear practical link between the health and education sectors.

Additionally, engagement with stakeholders through the extensive consultation and review processes ensured the workforce projections developed were relevant, trusted and supported across the sector. This has meant HW 2025 results have been accepted as an evidence base upon which policy decisions are made.

Finally, the iterative nature of HW 2025 provides a means for the impact of incremental adjustments to the health workforce to be measured, taking into account significant changes in the health system or the underlying social and economic environment. This, along with the alignment of student and training intakes to projected health workforce requirements, is vital in avoiding previous boom and bust cycles of supply of the health workforce.

## Results

Summary results from the HW 2025 project are presented for doctors, nurses and medical specialties. Additional results were generated for midwives, registered nurses and enrolled nurses, and registered nurses and enrolled nurses by area of practice^d^. These results are available in the HW 2025 suite of publications [14-16].

### Doctors

**Figure 2:**
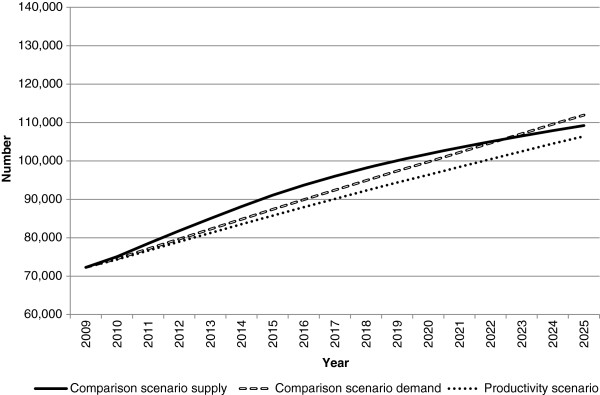
**Medical workforce supply and demand projections: productivity scenario.** This illustrates the potential impact of productivity improvements on medical workforce requirements relative to the comparison scenario. This was modelled by reducing workforce demand at a notional rate of 5% over the projection period.

**Figure 3:**
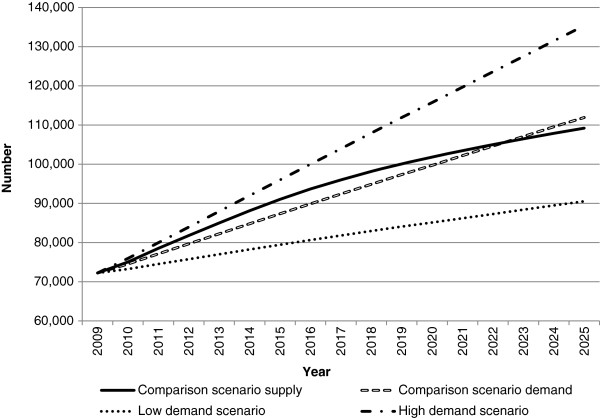
**Medical workforce supply and demand projections: high and low demand scenarios.** This illustrates the potential impact of changes in demand on future medical workforce requirements relative to the comparison scenario. In the low demand scenario, the demand for the workforce was reduced by a notional value of two percentage points. In the high demand scenario, the demand for the workforce was increased by a notional value of two percentage points.

**Figure 4:**
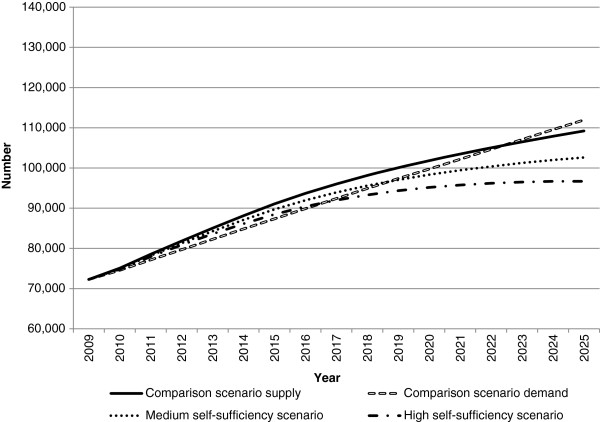
**Medical workforce supply and demand projections: medium and high self-sufficiency scenarios.** This illustrates the potential impact of changes in immigration levels on future medical workforce requirements relative to the comparison scenario. In the medium and high self-sufficiency scenarios, immigration was progressively reduced to 50% and 95% of starting levels, respectively.

Figures 2, 3 and 4 present the workforce supply and demand projections for the comparison and alternative scenarios for the medical workforce. The comparison scenario indicates that if current trends and conditions were to continue into the future, the medical workforce would largely be sustainable without changes to policy settings, with workforce demand exceeding supply by approximately 2,700 doctors in 2025.

Both innovation and reform scenarios (productivity and low demand) have a positive impact on the workforce gap relative to the comparison scenario (Figures 2 and 3). Under these scenarios, the medical workforce moves from a position of demand exceeding supply in 2025 under the comparison scenario to supply exceeding demand - by approximately 2,800 doctors in the productivity scenario, and 18,700 doctors in the low demand scenario. While both scenarios do not attribute their effects to particular measures, they demonstrate the potential aggregate effects of achieving specific improvements in productivity, or lowering demand for the medical workforce.

The self-sufficiency scenarios reduce workforce supply by reducing the number of migrants. Both self-sufficiency scenarios result in workforce demand substantially exceeding workforce supply in 2025, by approximately 9,300 doctors for medium self-sufficiency and 15,200 under high self-sufficiency (Figure 4). In both scenarios, demand exceeds supply earlier than the comparison scenario - in 2017 for medium self-sufficiency and 2019 for high self-sufficiency. These results demonstrate the significant role of international contributions to the medical workforce in meeting current and projected future demand.

Of all scenarios modelled, the high demand scenario has the greatest impact relative to comparison scenario - with demand exceeding supply by approximately 26,000 doctors (Figure 3). Reasons for increasing demand could include changing community expectations and increases beyond those predictable by effects such as aging and burden of disease. This highlights that any increases in demand (with other factors remaining the same) would have a substantial impact on the requirement for doctors.

### Nurses

**Figure 5:**
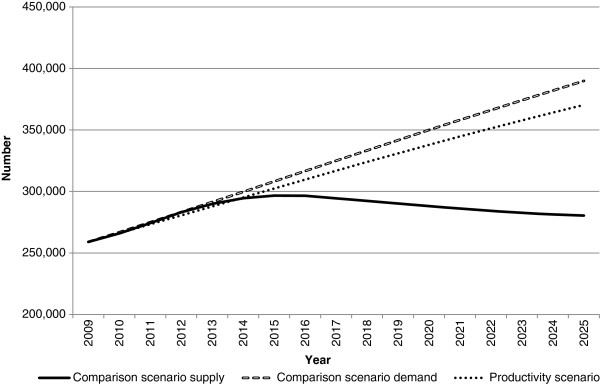
**Nursing workforce supply and demand projections: productivity scenario.** This illustrates the potential impact of productivity improvements on nursing workforce requirements relative to the comparison scenario. This was modelled by reducing workforce demand at a notional rate of 5% over the projection period.

**Figure 6:**
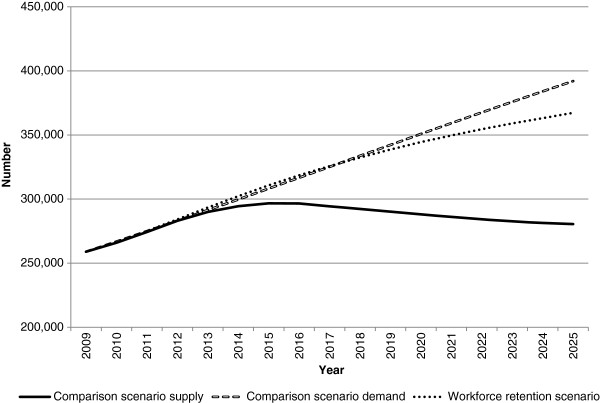
**Nursing workforce supply and demand projections: workforce retention scenario.** This illustrates the potential impact on nursing workforce supply of retaining nurses in the workforce. This was modelled by retaining 2007/2008 nursing exit rates (which were substantially lower than historical levels) across the projection period.

**Figure 7:**
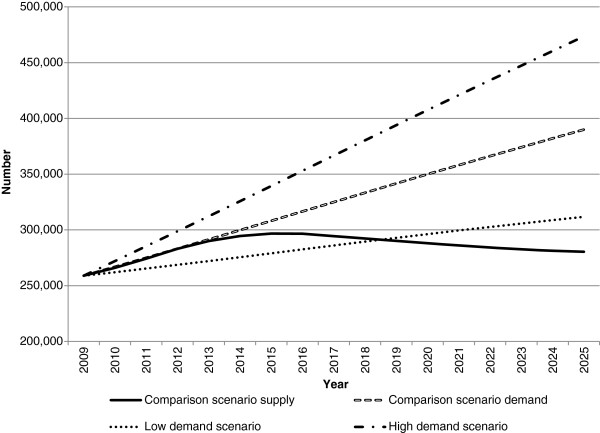
**Nursing workforce supply and demand projections: high and low demand scenarios.** This illustrates the potential impact of changes in demand on future nursing workforce requirements relative to the comparison scenario. In the low demand scenario, the demand for the workforce was reduced by a notional value of two percentage points. In the high demand scenario, the demand for the workforce was increased by a notional value of two percentage points.

**Figure 8:**
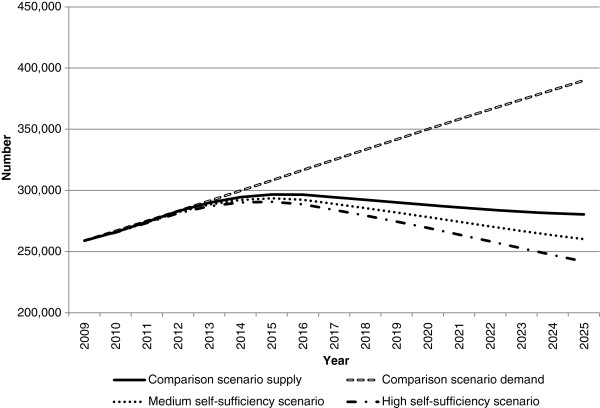
**Nursing workforce supply and demand projections: medium and high self-sufficiency scenarios.** This illustrates the potential impact of changes in immigration levels on future nursing workforce requirements relative to the comparison scenario. In the medium and high self-sufficiency scenarios, immigration was progressively reduced to 50% and 95% of starting levels, respectively.

Figures 5, 6, 7 and 8 present the workforce supply and demand projections for the comparison and alternative scenarios for the nursing workforce.

In developing the nursing workforce projections, examination of recent trends showed nursing exit rates for the period 2007/2008 were markedly lower than those from 2001 to 2006 (likely a result of the impact of the tighter economic environment on superannuation savings). For the comparison scenario, where recent trends are assumed to continue into the future, an informed decision was made (using the consultation and review processes outlined earlier) to apply the 2007/2008 exit rates until 2012, after which they reverted in equal increments to the 2001 to 2006 levels, until from 2016 onwards the 2001 to 2006 exit rates applied fully.

In the comparison scenario, a significant nursing workforce gap is projected without change to policy settings, with the exit rates reverting to 2001 to 2006 levels from 2016. The comparison scenario estimates the demand for nurses will exceed supply from approximately 2014 onwards, with a shortfall of almost 110,000 nurses by 2025.

Each of the innovation and reform scenarios (productivity - Figure 5; workforce retention - Figure 6; and low demand - Figure 7) reduce the amount by which the demand for nurses exceeds supply relative to the comparison scenario in 2025. Of the three innovation and reform scenarios, the workforce retention scenario has the greatest impact in reducing the gap between nursing workforce demand and supply in 2025 (Figure 6). In this scenario, the nursing exit rates observed in 2007/2008 (which were substantially lower than historical levels) were retained across the projection period other than through normal ageing effects. This demonstrates the sensitivity of the model to the nursing exit rate, and provides insight into the effects of retention strategies on meeting the demand for nurses.

The self-sufficiency scenarios extend the amount by which workforce demand exceeds supply relative to the comparison scenario; however, the impact is not as significant when compared with the results for doctors (Figure 8). The impact of the self-sufficiency scenario for nurses is also not as substantial as the impact of the innovation and reform scenarios, indicating the nursing workforce is not overly sensitive to changes in immigration.

### Medical specialties

Table 2 provides a summary of selected medical specialty workforce projections, showing the net difference between projected workforce supply and expressed demand in 2025 under each alternative scenario. Where the difference is positive, workforce supply increased relative to workforce demand; where negative, expressed demand increased relative to workforce supply. Workforce supply in 2009 is also shown to indicate the magnitude of the movement under each scenario.

**Table 2:** Selected medical specialty results - net workforce movement (headcount), 2025

			**Net workforce movement in 2025 (difference between workforce supply and expressed demand)**			
**Medical specialty**	**Existing workforce position**	**2009 workforce supply**	**Comparison scenario**	**Service and workforce reform scenario**	**Medium self-sufficiency scenario**	**Capped working hours scenario**
Anaesthesia	Orange	3,476	130	861	-71	85
Emergency medicine	Orange	1,134	-40	221	-138	-80
General practice	Red	26,389	57	6,590	-3,831	8
Intensive care	Green	517	35	184	9	-96
Obstetrics and gynaecology	Orange	1,562	-142	221	-302	-265
Ophthalmology	Orange	843	-162	28	-204	-180
Psychiatry	Red	2,981	-452	321	-784	-498
Radiation oncology	Red	245	-57	25	-65	-91
General surgery	Orange	1,181	519	829	430	296
Orthopaedic surgery	Green	1,168	148	444	90	7

In addition to the workforce projection results, Table 2 also shows the existing workforce position (EWP) assessment of selected medical specialties. (Results are not presented for all medical specialties that workforce projections were generated for. All medical specialty results are contained in [16].) For the medical and nursing workforce projections, it was assumed that the workforce was in balance at the beginning of the projection period. Feedback from stakeholders indicated this was not realistic, so prior to the development of the medical specialty workforce projections (which occurred after publication of the medical and nursing workforce projections), the EWP assessment was developed.

The EWP provides context for interpreting the workforce projection results, rather than assuming the workforce projections started from a position of balance. The EWP was determined from expert opinion from Australian state and territory government health departments, private employers and the profession, and an analysis of current vacancies and waiting times (where relevant and available). The EWP scale was:

Green: no current perceived shortage - sufficient workforce for existing expressed service demand, minimal number of vacancies, no difficulty filling positions, and short waiting times.

Orange: some level of expressed demand exceeding available workforce - either through mal-distribution or insufficient workforce numbers, some vacancies exist, with difficulty in filling positions.

Red: perceived current shortage - expressed service demand in excess of existing workforce, ongoing vacancies exist, difficult/unable to fill positions, and extended waiting times.

The EWP assessment identified that imbalances exist across the medical specialty workforces. While some medical specialties received an EWP assessment of green (no current perceived shortage), most were assessed as orange (perceived to have some level of expressed demand exceeding available workforce), and some were assessed as red (currently in shortage, with expressed service demand exceeding the existing workforce). Specialties perceived to be in shortage included general practice, general medicine, medical oncology, psychiatry, and radiation oncology [16].

For the medical specialties, the workforce projection results should be interpreted relative to the EWP assessment. Where workforce supply increases relative to demand (that is, the net workforce movement in Table 2 is positive), this does not necessarily imply a workforce will be in oversupply in 2025, particularly where the EWP assessment is red or orange. Key findings from the medical specialty workforce projections were: ongoing imbalances between medical specialties if current trends and conditions were to continue into the future; the service and workforce reform scenario (which incorporates a combination of reducing demand and increasing workforce productivity) had the greatest positive workforce impact relative to the comparison scenario of all the alternative scenarios; and the impact of the medium self-sufficiency scenario varied by medical specialty, demonstrating some workforces are more reliant on immigration.

## Discussion

The findings from HW 2025 highlighted a ‘business as usual’ approach to Australia’s health workforce is not sustainable over the next 10 years, with a need for co-ordinated, long-term reforms by government, professions and the higher education and training sector for a sustainable and affordable health workforce. The main policy levers identified to achieve change were innovation and reform, immigration, training capacity and efficiency and workforce distribution.

In particular, innovation and reform was highlighted as essential to a sustainable, affordable health workforce. In their 2005 report on Australia’s health workforce, Australia’s Productivity Commission^e^ noted that “productivity-enhancing improvements to health workforce arrangements are critical to ensuring a sustainable health care system, particularly given the constraints on government funding for health care” [17]. The innovation and reform scenarios in HW 2025 support this, demonstrating a substantial impact on projected workforce requirements. In Australia, recent workforce reforms have focussed on primary health care delivery, including support for new roles such as nurse practitioners by enabling access to Medicare (Australia’s universal health insurance scheme which provides access to free or subsidised treatment by authorised practitioners). HWA’s workforce innovation and reform programme is also supporting workforce reforms through expanded scopes of practice projects (such as expanding the use of physiotherapists in emergency departments and extending the role of paramedics) and building the role of rural medical generalists.

From the HW 2025 results, five policy proposals relating to the levers of innovation and reform, immigration, training capacity and efficiency and workforce distribution were approved by SCoH: 1) improved productivity through workforce innovation and reform; 2) improved mechanisms for the provision of efficient training; 3) addressing barriers and enablers to workforce reform; 4) streamlining clinical training funding; and 5) considerations for achieving national self-sufficiency.

From these five proposals, HWA is currently pursuing two work programmes specifically relevant to the retention and distribution of the health workforce. Under the first proposal (improved productivity through workforce innovation and reform), the Nursing Retention and Productivity Project is being progressed, and under the second proposal (improved mechanisms for the provision of efficient training) the National Medical Training Advisory Network (NMTAN) is being established.

### Nursing Retention and Productivity Project

For nurses, the workforce retention scenario had the greatest impact on the nursing workforce, demonstrating that improving the retention rate and keeping nurses in the workforce is an effective option in minimising potential future workforce shortages.

This project will propose a set of recommendations for nationally co-ordinated action by government, industry, the higher education sector, and national nursing organisations to improve nurse workforce retention and productivity. The project and recommendations were informed by: individual meetings with key stakeholders including clinical, jurisdictional and non-government representatives in each Australian State and Territory; a consultation document which received 84 submissions from organisations and individuals; a key stakeholder workshop, with over 80 representatives; a call for stories from nursing students and recent graduates on their experiences and expectations; a literature scan identifying key national and international innovations and reforms in nursing retention and productivity; and a project advisory group.

The recommendations have been developed and are due to be presented to SCoH in April 2014.

If fully implemented, the recommendations will help to develop and maintain a sustainable, flexible, skilled nursing workforce to deliver safe, effective care within a multi-disciplinary team.

### National Medical Training Advisory Network

Australia has no national co-ordinating mechanism linking vocational training availability for each medical specialty with the workforce needs of the community. Consequently, supply of each specialty group has been driven by factors not directly related to the community’s requirement for health services, including: trainee career preferences; the service requirements for trainees - that is, the reliance on trainees rather than specialists to provide services within parts of the health system; and the remuneration opportunities of different specialties.

NMTAN is being designed to generate policy advice that improves co-ordination of medical training to meet Australia’s workforce need. NMTAN will enhance planning, co-ordination and governance of medical training from profession entry through to vocational training by: aligning medical training effort with agreed national workforce requirements, focused on those areas where national effort adds value in addressing identified issues; progressing targeted medical training reforms, including those addressing geographic mal-distribution; forging stronger links between medical training activity, the health needs of the community and emerging models of care; and providing expert policy advice and guidance to the government, higher education, training and regulation sectors on national medical training issues.

The primary product from NMTAN is a series of rolling medical training plans with a focus on better co-ordination of medical education.

From these plans, NMTAN will identify annual target ranges for: medical student intakes; internships; basic and advanced trainee positions by specialty; and immigration requirements.

These targets will be reported to SCoH. Subject to the availability and robustness of data, these estimates will provide a level of geographic analysis to a state level (for smaller Australian states and territories) and at a regional level for larger Australian states.

NMTAN is in its establishment phase, and in 2013/2014 the concept of operations will be developed and implemented. The first national medical training plan is also due to be delivered to SCoH in the second quarter of 2014.

## Conclusion

HW 2025 workforce projections provided Australia’s first major, long-term national projections for the health workforce to 2025. The projections were developed using a principles and evidence-based approach, and demonstrated that a ‘business as usual’ approach to Australia’s health workforce is not sustainable over the next 10 years.

The evidence basis upon which the workforce projections were developed enabled them to be used as a framework for a nationwide discussion on future directions for workforce policy and reform directions. From the policy proposals presented to SCoH, HWA is actively pursuing two work programmes that relate directly to key findings from the workforce projections. These work programmes will inform policy development relating to the productivity and retention of Australia’s nursing workforce, and the distribution of the medical workforce across medical specialties, to best match community health needs.

HW 2025 is also not a one-off project. It is an ongoing process where HWA will continue to develop and improve health workforce projections incorporating data and methodology improvements to support incremental health workforce changes.

## Endnotes

^a^Australia has a federal system of government, where powers are divided between a national government and state and territory governments.

^b^COAG is the peak intergovernmental forum in Australia. COAG promotes policy reforms that are of national significance, or which need co-ordinated action by all Australian Governments. COAG is supported by ongoing standing councils.

^c^SCoH is one of the standing councils supporting COAG. It is comprised of Australian Commonwealth, State, Territory and New Zealand Ministers with responsibility for health matters, and the Commonwealth Minister for Veteran’s affairs.

^d^There are two levels of regulated nurses in Australia - Registered Nurses (RNs) and Enrolled Nurses (ENs). A RN is a person who has completed, as a minimum, a 3-year bachelor degree and is registered with the Nursing and Midwifery Board of Australia (NMBA). RNs practise independently and interdependently, assuming accountability and responsibility for their own actions and delegation of care to ENs and other health care workers. An EN usually works with RNs to provide patients with basic nursing care, doing less complex procedures than RNs. ENs must complete accredited training through a Vocational Education Training provider, and are also registered with the NMBA. In HW 2025, workforce projections were developed for RNs and ENs, as well as RNs and ENs categorised to the following areas of practice: acute care, critical care and emergency, aged care, mental health and all other areas.

^e^The Productivity Commission is the Australian Government's independent research and advisory body on a range of economic, social and environmental issues affecting the welfare of Australians.

## Abbreviations

AHWAC: Australian Health Workforce Advisory Committee; AMWAC: Australian Medical Workforce Advisory Committee; COAG: Council of Australian Governments; EN: Enrolled Nurse; EWP: existing workforce position; NMBA: Nursing and Midwifery Board of Australia; HW 2025: Health Workforce 2025; HWA: Health Workforce Australia; NMTAN: National Medical Training Advisory Network; RN: Registered Nurse; SCoH: Standing Council on Health.

## Competing interests

The authors declare that they have no competing interests.

## Authors’ contributions

IFC was the project lead and, in conjunction with MVM as project manager, designed the project and the alternative planning scenarios. MVM was responsible for conducting the workforce modelling. BJF designed and drafted the manuscript. TH and MCT assisted with conducting the workforce modelling and were involved in drafting of the manuscript. ST assisted with the workforce modelling. All authors read and approved the final manuscript.

## Authors’ information

All authors are employed by Health Workforce Australia, the organisation which conducted the workforce projections and is funding and managing the Nursing Productivity and Retention Project and the National Medical Training Network, and is the organisation that is financing the manuscript.
